# Association Between Air Pollution and the Risk of Uveitis: A Nationwide, Population-Based Cohort Study

**DOI:** 10.3389/fimmu.2021.613893

**Published:** 2021-03-18

**Authors:** Yi-Chiao Bai, Cheng-You Wang, Cheng-Li Lin, Jung-Nien Lai, James Cheng-Chung Wei

**Affiliations:** ^1^Institute of Medicine, Chung Shan Medical University, Taichung, Taiwan; ^2^Department of Optometry, Shu-Zen Junior College of Medicine and Management, Kaohsiung, Taiwan; ^3^College of Medicine, Chung Shan Medical University, Taichung, Taiwan; ^4^Institute of Medicine, E-DA Hospital, Kaohsiung, Taiwan; ^5^Management Office for Health Data, China Medical University Hospital, Taichung, Taiwan; ^6^School of Chinese Medicine, College of Chinese Medicine, China Medical University, Taichung, Taiwan; ^7^Department of Chinese Medicine, China Medical University Hospital, Taichung, Taiwan; ^8^Division of Allergy, Immunology and Rheumatology, Department of Internal Medicine, Chung Shan Medical University Hospital, Taichung, Taiwan; ^9^Graduate Institute of Integrated Medicine, China Medical University, Taichung, Taiwan

**Keywords:** air pollution, uveitis, risk factor, urbanization, inflammation

## Abstract

Previous studies have revealed an association between ocular surface disorders and air pollution, few studies have focused on the risk of uveitis. We aimed to investigate whether air pollution increases the risk of uveitis. We used the Taiwan Longitudinal Health Insurance Database (LHID) and the Taiwan Air Quality Monitoring Database (TAQMD) to conduct a retrospective cohort study. Air pollutant concentrations, including those of carbon dioxide (CO_2_), were grouped into four levels according to quartiles. The outcome was the incidence of uveitis, as defined in the International Classification of Diseases, Ninth Revision. We used univariable and multivariable Cox proportional hazard regression models to calculate the adjusted hazard ratios (aHRs) and determine the potential risk factors of uveitis. Overall, 175,489 subjects were linked to their nearby air quality monitoring stations. We found that for carbon monoxide, the aHRs of uveitis risk for the Q3 and Q4 levels were 1.41 (95% confidence interval (CI) = 1.23–1.61) and 2.19 (95% CI = 1.93–2.47), respectively, in comparison with those for the Q1 level. For nitric oxide, the aHRs for the Q3 and Q4 levels were 1.46 (95% CI = 1.27–1.67) and 2.05 (95% CI = 1.81–2.32), respectively. For nitrogen oxide (NOx), the aHRs for the Q2, Q3, and Q4 levels were 1.27 (95% CI = 1.11–1.44), 1.34 (95% CI = 1.16–1.53), and 1.85 (95% CI = 1.63–2.09), respectively. For total hydrocarbon (THC), the aHRs for the Q2, Q3, and Q4 levels were 1.42 (95% CI = 1.15–1.75), 3.80 (95% CI = 3.16–4.57), and 5.02 (95% CI = 4.19–6.02), respectively. For methane (CH4), the aHRs for the Q3 and Q4 levels were 1.94 (95% CI = 1.60–2.34) and 7.14 (95% CI = 6.01–8.48), respectively. In conclusion, air pollution was significantly associated with incidental uveitis, especially at high THC and CH_4_ levels. Furthermore, the uveitis risk appeared to increase with increasing NOx and THC levels.

## Introduction

Uveitis is an inflammatory and sight-threatening disorder that contributes to 2.8%–10% of all cases of blindness (1–4). It also aggravates pain and decreases visual function, thereby affecting the quality of life of patients, and may also cause socio-economic impacts at the country level (5, 6). Past studies have found possible ocular complications related to uveitis, such as corneal deposition, cataracts, secondary glaucoma, macular edema, vasculitis, and retinitis (7). Indeed, the classification of subtypes of uveitis is divided into four anatomical categories—anterior uveitis, middle uveitis, posterior uveitis, and panuveitis—by the International Uveitis Research Group (IUSG) (8, 9). Of the total, previous reports showed that anterior uveitis accounted for 24.5%–52.3%, panuveitis accounted for 11.8%–52.9%, posterior uveitis accounted for 7.1%–46.0%, and the prevalence of intermediate uveitis accounted for 6.3%–19.3%; furthermore, anterior uveitis is the most common form of uveitis in Asia (10, 11). The types of inflammation associated with uveitis can be divided into infectious and non-infectious causes. Moreover, non-infectious uveitis, including immune-mediated diseases, may be related to other systemic diseases, or partial makeup syndrome, such as ankylosing spondylitis, Behcet’s disease, sarcoidosis, VKH disease, and juvenile idiopathic arthritis (12–15). The cause of uveitis may be infectious, such as viruses, bacteria, parasites, and fungi. In the United States, the estimated prevalence of non-infected uveitis is 121 cases per 100,000 (95% CI, 117.5–124.3) and 29 per 100,000 for children (95% CI, 26.1–33.2). The degree of urbanization of a country also affects the cause of uveitis and varies greatly. Epidemiological studies of uveitis have found that the cumulative incidence of uveitis has risen in Taiwan, increasing from 318.8 cases per 100,000 people in 2003 to 622.7 cases per 100,000 people in 2008, and also have found that the population living in urban areas is most affected by uveitis, particularly the elderly and those living alone (16). Previous studies have found that both genetic and environmental factors may affect uveitis (17). However, data on the impact of air pollution on uveitis in clinical settings are limited.

Air pollution, a widespread, single environmental risk factor, has gradually attracted the attention of many researchers owing to its adverse health consequences in various body systems (18), including systemic autoimmune disease (19), intestinal disease (20), type 2 diabetes mellitus (21), and cardiovascular diseases (22), to which increased inflammatory cytokine production is related. The World Health Organization (WHO) previously showed that 58% of air-pollution-related mortality was attributed to cardiovascular disease, 18% to chronic obstructive pulmonary disease (COPD) and acute lower respiratory tract infections, and 6% to lung cancer (23). Air pollution can be divided into two categories, natural phenomena and human activities, and man-made air pollution sources can result in the most harmful adverse health effects; these sources include carbon monoxide (CO) from automobile exhaust, nitrogen oxides (NOx), or sulfur dioxide (SO2) from industrial processes. Pollutants, including particulate matter (PM), ozone (O_3_), nitrogen dioxide (NO_2_), and sulfur dioxide (SO_2_), have been proven to negatively affect health with long-term exposure. A global investigation revealed that an estimated 4.2 million premature deaths are believed to be associated with ambient air pollution (24). Several studies have also shown that ocular surface inflammation and dry eye disorder are related to high concentrations of air pollution (25–28). Although several studies have investigated the association between ocular surface disorder and air pollution, few have focused on the impact of air pollution on the uvea. Accordingly, our study aimed to investigate the relationship between exposure to common air pollutants and uveitis in a nationwide retrospective cohort study using the National Health Insurance Research Database (NHIRD).

## Materials and Methods

### Data Source

**Figure 1:**
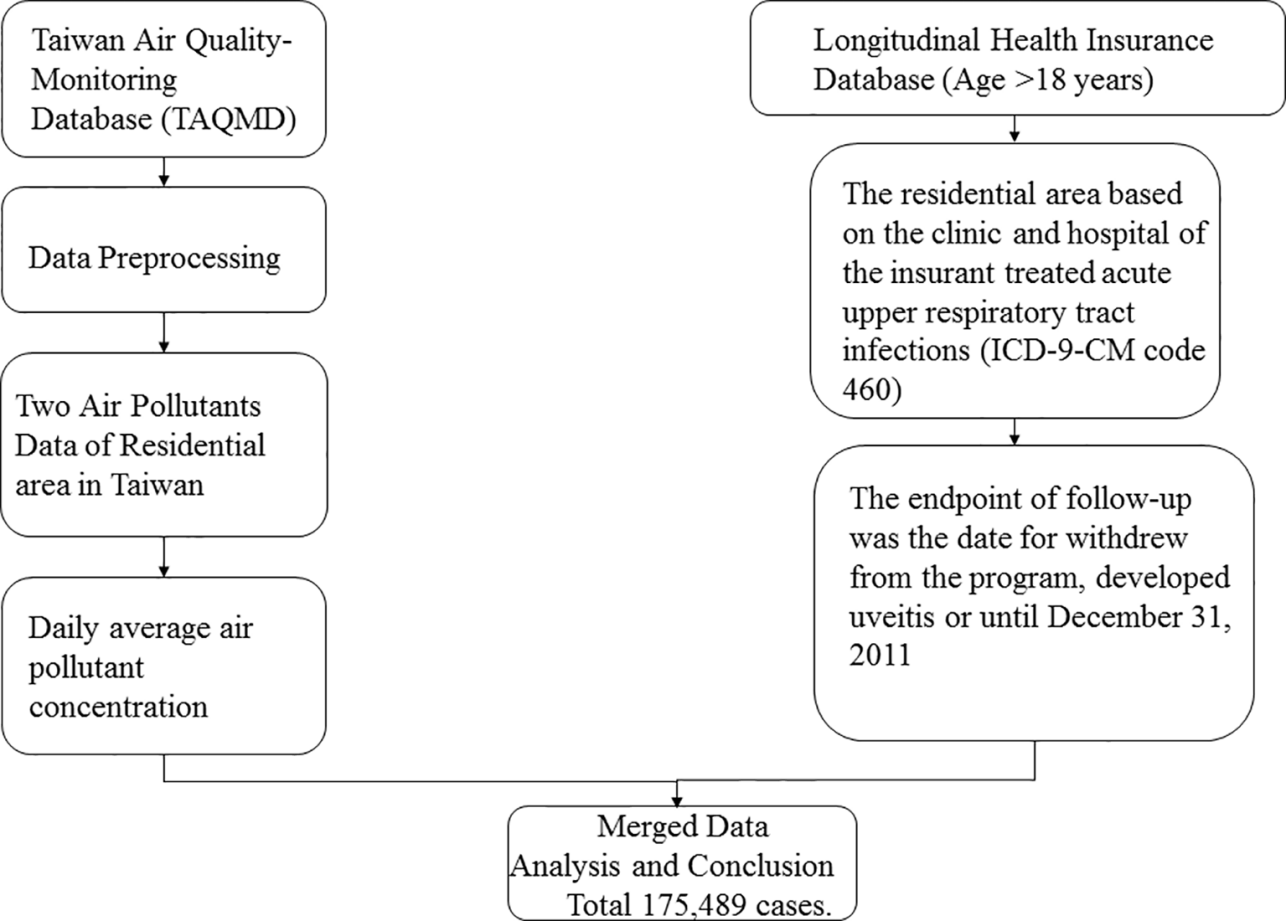
Flow chart of inclusion criteria.

We enrolled participants from the National Health Research Insurance database (NHIRD) between 2000 and 2010. Taiwan launched a single-payer National Health Insurance (NHI) program in 1995, and 99.9% of Taiwan’s population was enrolled. This cohort study used the Taiwan Longitudinal Health Insurance Database (LHID) 2000, a part of the NHIRD. LHID 2000 comprises 1,000,000 randomly sampled beneficiaries enrolled in the National Health Insurance (NHI) program. Data in the registry of beneficiaries comprise a unique encrypted identifier, sex, date of birth, and an insured payroll-related amount. The claims data also contain diagnoses, prescriptions, and details of inpatient care or outpatient visits. Disease diagnoses were coded using the International Classification of Diseases, Ninth Revision (ICD-9 CM code). Although medical facilities have been de-identified, their residential area code can be obtained from the registry (**Figure 1**).

Air pollution data were retrieved from the Taiwan Air Quality Monitoring Database (TAQMD), which has gathered information from 78 air quality monitoring stations in Taiwan since 1993. Most monitoring stations are located in populous urban and rural areas and can be used to inform the public on ambient air quality. Open data on hourly measurements of major pollutants are available on the monitoring network website. These hourly data recorded at each TAQMD were further averaged into daily mean concentrations in this study. Air pollutant monitoring stations routinely monitor several pollutant levels, including those of nitrogen oxide (NOx), carbon oxide, and particulate matter (PM), in addition to weather conditions, such as temperature and humidity (**Figure 1**).

### Sample Participant, Outcome, and Exposure Measurement

This retrospective observational study was conducted between January 1, 2000 and December 31, 2011 using the Taiwan Health Insurance Database. All recruited participants were aged ≥20 years. The binary outcome variable was the occurrence of uveitis *(*ICD-9-CM codes 360.00, 360.11, 360.12, 362.18, 363.00, 363.01, 363.03, 363.05 363.08, 363.1x, 363.20, 363.21, 363.4x, 364.00 364.02, 364.04, 364.1x, and 364.3x) and the major predictive variables included the exposure levels to carbon monoxide (CO), nitric oxide (NO), NOx, total hydrocarbon (THC), and methane (CH_4_). The participant exposure was based on the exposure of the station where the participants resided. The air pollutant monitoring station was based on the nearest station. The daily average cumulative exposure of the participant was observed from 2000 to the end point. The cumulative exposure per hour was multiplied by 24 as the basis for daily exposure, which was excluded if there was more than 8 hours of observed omissions. The daily average air pollutant concentration was estimated and considered as the concentration based on the data from the TAQMD. We used quartiles as the basis for grouping exposure concentrations. To corroborate the exposure–response relationship, we categorized the daily concentrations of CO, NO, NOx, THC, and CH_4_ into the following quartiles: <25th percentile (Q1), 25–50th percentile (Q2), 50–75th percentile (Q3), and >75th percentile (Q4). In this study, we use Inverse Distance Weighting (IDW) methods to analysis the sensitivity analysis. In IDW method, it is assumed substantially that the rate of correlations and similarities between neighbors is proportional to the distance between them that can be defined as a distance reverse function of every point from neighboring points. It is necessary to remember that the definition of neigh-boring radius and the related power to the distance reverse function are considered as important problems in this method. These methods will be used by a state in which there are enough sample points (at least 14 points) with a suitable dispersion in local scale levels (**Supplement Table 1**).

The confounding factors were age, sex, urbanization level of residence, and comorbidities, including diabetes mellitus *(*ICD-9-CM code 250), hypertension (ICD-9-CM codes 401-405), hyperlipidemia (ICD-9-CM code 272), asthma (ICD-9-CM code 493), COPD (ICD-9-CM codes 490-496), psoriatic disease (ICD-9-CM code 696), rheumatoid arthritis (ICD-9-CM code 714), SLE (ICD-9-CM code 710.0), and Behcet’s syndrome (ICD-9-CM code 136.1). Residential areas were classified into four levels: level 1 representing the area with the highest urbanization level and level 4 representing the area with the lowest urbanization level. All participants were followed up from January 1, 2000 until the diagnosis of uveitis, withdrawal from the NHI program, or December 31, 2011.

### Statistical Analysis

We calculated the percentage of the participants in this study by sex, age, urbanization level, and comorbidities. We also calculated the mean values and standard deviations (SDs) of the CO, NO, NOx, THC, and CH_4_ concentrations. The chi-squared test was used to compare the differences in the urbanization level in each quartile of the daily average concentration of air pollutants. Furthermore, we treated each air pollutant concentration as a categorical variable and reported the hazard ratio (HR) per interquartile range (IQR) increase (μg/m^3^ or ppb). The covariates recorded in this study included sex, age, urbanization level, and comorbidities during the study period. Cox proportional hazard regression models were applied to evaluate the associations between exposure to ambient air pollution during the study period and the incidence of uveitis. The relative risk of uveitis in the participants who were exposed to the Q2–Q4 levels of air pollutants in relation to that in those who were exposed to the Q1 level was assessed using the Cox proportional hazard regression model. Kaplan–Meier curves estimating the cumulative incidence of uveitis among the IQR groups according to the CO, NO, NOx, THC, and CH_4_ concentrations were applied. All statistical analyses were performed using the SAS statistical software, and the significance level was set at p-values of <0.05, based on a two-tailed calculation. The **Supplement Table 2** showed that exposure were categorized into 3 groups based on tertile and stratify by follow-up period to estimate the hazard ratios of Uveitis as sensitivity testing.

## Results

### The Characteristics of the Participants and Air Pollutant Concentrations

**Table 1** demonstrates the basic characteristics of the participants. In total, 175,489 participants aged ≥20 years were selected from a nationally representative sample from the Taiwan LHID from 2000 to 2011. The cohort had a mean age (SD) of 39.6 ± 15.4 years. Among all subjects, insured women (55.9%) and those living in urbanization level 1 (34.2%) accounted for the largest percentage. The mean daily air pollutant concentrations were 0.72 ± 0.27 ppb for CO, 11.0 ± 10.1 ppb for NO, 36.3 ± 34.9 ppb for NOx, 2.41 ± 0.23 ppm for THC, and 2.02 ± 0.13 ppm for CH_4_. A total of 2205 patients were diagnosed with uveitis during the follow-up period of 11.5 years.

**Table 1:** Baseline demographics and exposure to air pollutants by yearly average concentration in Taiwan, 2000–2011.

N = 175,489		n	%
Gender	Female	98,074	55.9
	Male	77,415	44.1
Age, years	mean, SD	39.6	15.4
Urbanization level^†^	1 (highest)	60,056	34.2
	2	56,889	32.4
	3	29,940	17.1
	4 (lowest)	28,603	16.3
Comorbidity			
Diabetes		19,498	11.1
Hypertension		54,465	31.0
Hyperlipidemia		46,057	26.2
Asthma		21,027	12.0
COPD		39,041	22.3
Psoriatic diseases		2837	1.62
RA		512	0.29
SLE		197	0.11
Behçet’s syndrome		16	0.01
Exposure of air pollutants			
CO level (yearly average, ppm)	mean, SD	0.72	0.27
NO level (yearly average, ppb)	mean, SD	11.0	10.1
NOx level (yearly average, ppm)	mean, SD	36.3	34.9
THC (yearly average, ppm)	mean, SD	2.41	0.23
CH4 (yearly average, ppm)	mean, SD	2.02	0.13
Outcome			
Uveitis	Yes	2205	1.26
Follow-up time, years	mean, SD	11.5	1.59

^†^The urbanization level was categorized according to the population density of the residential area into four levels, with level 1 as the most urbanized and level 4 as the least urbanized.

CO, carbon monoxide; NO, nitric oxide; NOx, nitrogen oxide; THC, total hydrocarbon; CH_4_, methane; SD, standard deviation.

### The Urbanization Level Areas and Exposed Air Pollutant Concentrations

**Table 2** presents the distribution of the urbanization levels among the different quartiles of air pollutant levels. The participants exposed to the Q4 level of air pollutants, including CO, NO, NOx, THC, and CH_4_, mostly resided in urbanization level 1 areas. Approximately 45.5% of those with high CO concentrations, 50.0% of those with high NO concentrations, 45.7 of those with high NOx concentrations, 43.5% of those with high THC concentrations, and 32.7% of those with high CH_4_ concentrations resided in high urbanization level areas.

**Table 2:** Baseline urbanization level according to the quartiles of daily average concentration of air pollutants in Taiwanese, 2000–2011.

Air pollutant Concentration	Quartile 1 (Q1) (lowest)		Quartile 2 (Q2)		Quartile 3 (Q3)		Quartile 4 (Q4) (highest)		*p-value
N = 175,489	n	(%)	n	(%)	n	(%)	n	(%)	
**Carbon monoxide (CO)**									<0.001
Urbanization level									
1 (highest)	9324	15.5	13,538	23.8	7505	25.1	13,018	45.5	
2	10,624	17.7	20,156	35.4	6760	22.6	7419	26.0	
3	15,334	25.5	9711	17.1	9325	31.2	5231	18.3	
4 (lowest)	24,772	41.3	13,478	23.7	6345	21.2	2915	10.2	
**Nitric oxide (NO)**									<0.001
Urbanization level									
1 (highest)	8927	14.9	14,880	26.2	4628	15.5	14,301	50.0	
2	10,692	17.8	14,751	25.9	11,013	36.8	8007	28.0	
3	17,505	29.2	12,460	21.9	6126	20.5	3462	12.1	
4 (lowest)	22,932	38.2	14,798	26.0	8173	27.3	2832	9.90	
**Nitrogen oxides (NOx)**									<0.001
Urbanization level									
1 (highest)	9285	15.5	14,048	24.7	5194	17.4	13,064	45.7	
2	11,673	19.4	17,035	30.0	9288	31.0	8790	30.8	
3	11,859	19.8	13,568	23.9	8110	27.1	3291	11.5	
4 (lowest)	27,237	45.4	12,233	21.5	7347	24.5	3443	12.0	
**Total hydrocarbon (THC)**									
Urbanization level									
1 (highest)	9991	21.5	8833	21.6	7725	30.9	8696	43.5	
2	8123	17.5	12,751	31.1	5935	23.8	6387	31.9	
3	14,531	31.3	9031	22.0	6546	26.2	2972	14.9	
4 (lowest)	13,768	29.7	10,371	25.3	4765	19.1	1948	9.74	
**Methane (CH4)**									<0.001
Urbanization level									
1 (highest)	10,212	22.0	9152	22.3	8938	35.8	6533	32.7	
2	10,888	23.5	9581	23.4	5392	21.6	3739	18.7	
3	16,498	35.6	15,070	36.8	6796	27.2	4558	22.8	
4 (lowest)	8815	19.0	7183	17.5	3845	15.4	5173	25.9	

^*^chi-squared test.

The urbanization level was categorized according to the population density of the residential area into four levels, with level 1 as the most urbanized and level 4 as the least urbanized.

The daily average air pollutant concentrations were categorized into four groups according to the quartiles for each air pollutant.

### Long-Term Trends in Air Pollutant Concentrations and the Risk of Uveitis

**Table 3** shows the incidence of uveitis according to the air pollutant concentrations. We controlled for potential confounding factors, including sex, age, urbanization level, and comorbidities, and considered the subjects exposed to the Q1 level of air pollutants as a reference group. We found that Q4 air pollutant level exposure increased the risk of uveitis significantly. For CO, the Q3 (adjusted hazard ratio (aHR) = 1.41, 95% confidence interval (CI) = 1.23–1.61) and Q4 (aHR = 2.19, 95% CI = 1.93–2.47) concentrations were significantly associated with a higher risk of uveitis than the Q1 level. For NO, the Q3 (aHR = 1.46, 95% CI = 1.27–1.67) and Q4 (aHR = 2.05, 95% CI = 1.81–2.32) concentrations were also significantly associated with a higher risk of uveitis than the Q1 level. For NOx, the Q2 (aHR = 1.27, 95% CI = 1.11–1.44), Q3 (aHR = 1.34, 95% CI = 1.16–1.53), and Q4 (aHR = 1.85, 95% CI = 1.63–2.09) concentrations were significantly associated with a higher risk of uveitis than the Q1 level. For THC, the relative risks of uveitis for the Q2, Q3, and Q4 levels were 1.42 (95% CI = 1.15–1.75), 3.80 (95% CI = 3.16–4.57), and 5.02 (95% CI = 4.19–6.02), respectively, in comparison with those for the Q1 level. For CH_4_, the aHRs for the Q3 and Q4 levels were 1.94 (95% CI = 1.60–2.34) and 7.14 (95% CI = 6.01–8.48), respectively, in comparison with those for the Q1 level.

**Table 3:** Risk of uveitis in the patients exposed to various air pollutants stratified by the quartile of daily average concentration using Cox proportional hazard regression models.

		Event	IR	cHR	(95%CI)	aHR^†^	(95%CI)
**Carbon monoxide (CO)**							
Quartile 1, <0.56 ppm		416	8.31	Reference group		Reference group	
Quartile 2, 0.56-0.68 ppm		387	7.41	0.89	(0.78, 1.02)	0.96	(0.83, 1.10)
Quartile 3, 0.68-0.81 ppm		482	10.6	1.28	(1.12, 1.46)***	1.41	(1.23, 1.61)***
Quartile 4, >0.81ppm		897	16.6	1.99	(1.78, 2.24)***	2.19	(1.93, 2.47)***
**Nitric oxide (NO)**							
Quartile 1, <5.16 ppb		401	8.13	Reference group		Reference group	
Quartile 2, 5.16-8.58 ppb		457	8.91	1.10	(0.96, 1.25)	1.14	(0.99, 1.30)
Quartile 3, 8.58-11.5 ppb		479	10.5	1.29	(1.13, 1.47)***	1.46	(1.27, 1.67)***
Quartile 4, >11.5 ppb		867	15.6	1.92	(1.70, 2.16)***	2.05	(1.81, 2.32)***
**Nitrogen oxides (NOx)**							
Quartile 1, <23.4 ppm		401	8.37	Reference group		Reference group	
Quartile 2, 23.4-32.0 ppm		542	10.1	1.20	(1.06, 1.37)**	1.27	(1.11, 1.44)***
Quartile 3, 32.0-38.6 ppm		433	10.2	1.22	(1.06, 1.40)**	1.34	(1.16, 1.53)***
Quartile 4, >38.6 ppm		826	14.3	1.72	(1.52, 1.93)***	1.85	(1.63, 2.09)***
**Total hydrocarbon (THC)**							
Quartile 1, <2.28 ppm		150	3.61	Reference group		Reference group	
Quartile 2, 2.28-2.38 ppm		207	5.31	1.47	(1.19, 1.81)***	1.42	(1.15, 1.75)***
Quartile 3, 2.38-2.56 ppm		501	13.3	3.69	(3.07, 4.42)***	3.80	(3.16, 4.57)***
Quartile 4, >2.56 ppm		604	17.2	4.77	(3.99, 5.71)***	5.02	(4.19, 6.02)***
**Methane (CH4)**							
Quartile 1, <2.00 ppm		155	3.77	Reference group		Reference group	
Quartile 2, 2.00-2.04 ppm		132	3.78	1.00	(0.80, 1.27)	0.96	(0.76, 1.22)
Quartile 3, 2.04-2.10 ppm		371	7.42	1.97	(1.63, 2.37)***	1.94	(1.60, 2.34)***
Quartile 4, >2.10 ppm		804	29.5	7.85	(6.61, 9.32)***	7.14	(6.01, 8.48)***

The daily average air pollutant concentrations were categorized into four groups according to the quartiles for each air pollutant.

^†^Adjusted for age, sex, urbanization level, and comorbidities, including diabetes mellitus, hypertension, hyperlipidemia, asthma, COPD, psoriatic diseases, rheumatoid arthritis, SLE, and Behcet’s syndrome.

^*^p<0.05, ^**^p<0.01, ^***^p<0.001.

IR, incidence rate (per 1,000 person-years); cHR, crude hazard ratio; aHR, adjusted hazard ratio; CI, confidence interval.

**Figure 2:**
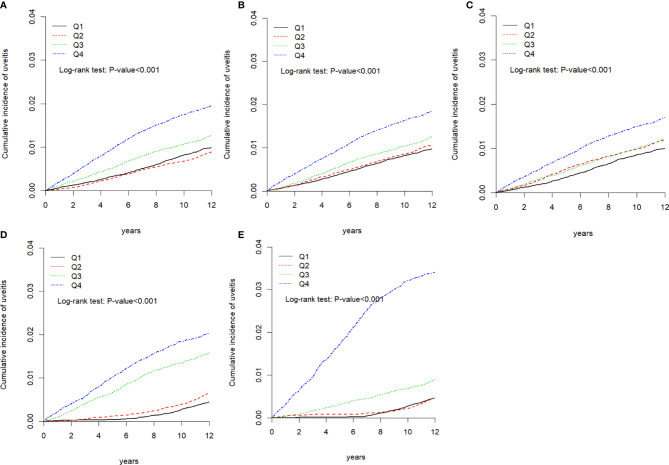
Kaplan-Meier curves of the cumulative incidence of uveitis during the follow-up period among the different quartiles of each air pollutant. **(A)** Carbon monoxide (CO), **(B)** nitric oxide (NO), **(C)** nitrogen oxide (NOX), **(D)** total hydrocarbon (THC), and **(E)** methane.

During the follow-up period, the cumulative incidence of uveitis in those with higher air pollutant exposure was significantly higher than that in those with lower air pollutant exposure (*p* < 0.001, **Figures 2A–E**). We computed the long-tern average exposure levels of these pollutants over 2-year periods before the diagnosis of uveitis, end of the study period for each individual. Then, we speculate the yearly concentration of air pollutants by IDW methods (**Supplement Table 1**). Furthermore, **Supplement Table 2** showed that the differences in uveitis incidences and associated HRs in participants ex-posed to daily average concentrations of CO, NO, NOx, THC and CH4 stratify by follow-up period.

## Discussion

This retrospective cohort study is the first study to associate uveitis with daily air pollution exposure in a large sample size from the aforementioned database. The analyses revealed that increasing quartile concentrations of CO, NO, NOx, THC, and CH4 were associated with an increasing risk of uveitis in Taiwan. Particularly with long-term exposure, a THC concentration of >2.56 ppm and a CH_4_ concentration of >2.10 ppm were associated with a significantly higher risk of uveitis than the other pollutant concentrations in the cohort (i.e., <2.28 ppm for THC and <2.00 ppm for CH_4_; THC group: aHR=5.02, 95% CI=4.19–6.02; CH_4_ group: aHR=7.14, 95% CI=6.01–8.48).

Several previous studies have already demonstrated that air pollution exposure can be significantly associated with ocular surface disorders or vascular conditions (29–31). However, the association between environmental factors and uveitis has not been discussed. Our study showed that the participants who were exposed to the Q4 level of air pollutants, including CO, NO, NOx, THC, and CH4, mostly resided in urbanization level 1 areas. The results are similar to those of studies that showed that air pollutants in metropolitan areas were more significantly associated with the urbanization level than those in rural areas (31, 32). Furthermore, the issue of air pollution has attracted the attention of researchers and the WHO; an increasing number of chronic systemic diseases can result from long-term exposure to air pollutants. Because this study considered seasonal changes in air pollution, we first calculated the per daily exposure. Then, we multiplied the average hourly exposure by 24 hours to obtain the mean daily air pollution concentrations, and then divided the air pollutant concentration into four levels based on the quartiles. The classification of urbanization levels in NHIRD is based on population density (persons/km^2^), with medical coverage per 100,000 people in each region. NHIRD data can be divided into seven levels of urbanization. We calculated the average air concentration of each patient’s exposure using the IDW method to estimate the air pollution concentration between the measured values of the air monitoring stations around the house registered by each patient based on the distance. The IDW method is one of the most commonly used spatial interpolation methods in the earth sciences. Our research results may provide warnings for long-term exposure to air pollution, and recommend strict monitoring of the quality of the air. Environmental pollution has an impact on the eyes, and uveitis can be caused by environmental risk factors. The clinical applicability of this study can provide public health-related information, such as measures to reduce exposure, the effectiveness of relevant health protection recommendations, and its impact on the health and well-being of the population.

The possible mechanisms of uveitis and air pollution remain unknown. It was hypothesized that air pollution could increase the incidence of uveitis. Uveitis is characterized by inflammation of the uvea and related eye structures of the vascular layer. The blood retinal barrier (BRB) is located in the retinal pigment epithelium (RPE) and the retinal vascular endothelium, which form the posterior and anterior barrier, respectively. This barrier function will restrict the entry of molecules, but in the process of eye inflammation, lymphocytes pass through the BRB and enter the retina in large quantities. It is conceivable that uveitis affects the inflammation of the retinal layer. The retina belongs to one part of the central nervous system (CNS), and loss of retina structure or function may play a key role in CNS diseases (33). Furthermore, several studies have noted that NOx pollution may cause inflammation, and induce lipid peroxidation and oxidative stress (34). Previous studies have found that exposure to CO and NO_2_ may be harmful to the eyes (35). However, the effects of THC and CH_4_ on eye diseases have almost been ignored in epidemiological studies. This study shows that exposure to THC and CH_4_ may have harmful effects on the eyes. In addition, the relationship between autoimmune diseases and various other diseases, including recognized diseases such as uveitis, and heart conduction problems, is related to AS. Furthermore, rheumatoid diseases can also lead to the development of uveitis, which indicates that uveitis and related inflammation can also damage eye tissue (36). In addition, other diseases associated with uveitis include Behcet’s disease, syphilis, seronegative spondyloarthritis, and psoriasis (9, 11). Extensive evidence has been reported with regard to the association between air pollution and autoimmune disease, which is believed to play a key role in the development of systemic inflammation. Van Eeden et al. also found elevated levels of inflammatory cytokines, such as IL-6, IL-1, and granulocyte macrophage colony-stimulating factor (GM-CSF), in individuals with long-term exposure to air pollution (37). Farhat et al. observed that environmental chemicals of air pollution contributed to autoimmune disease, suggesting that air pollution may increase the production of T lymphocytes (13). Picascia et al. observed that epigenetic modification-induced oxidative stress in relation to the pathogenesis of autoimmune diseases may be caused by exposure to an adaptive immune response, resulting in autoimmune syndrome and production of inflammatory cytokines (27).

Our study has several strengths. First, this is the first study to directly show the association between air pollution and uveitis using a nationwide database. Second, we analyzed a large sample of patients over 12 years, which yielded strong evidence on the long-term outcomes of this Taiwanese population. Third, because 99% of the Taiwanese population (around 23 million residents) is enrolled in the NHIRD, selection bias of region, age, and institution can be minimized. Fourth, we analyzed the trends of air pollutant concentrations in quartiles in relation to uveitis, which signifies a dose–response relationship; this can clearly be observed in our analysis between air pollution and uveitis. This study also indicates the air pollution exposure as a risk factor for uveitis. However, this cohort study also has some limitations. First, the LHID and TAQMD datasets were combined by the residential areas of the insurants linked to nearby air quality monitoring stations, and all participants’ residential areas were based on the clinic or hospital that the insurants attended to determine the exposure area in this study. Thus, the results could be misinterpreted by ignoring underlying confounders and baseline comparability. Second, the NHIRD does not record detailed information on alcohol consumption, socioeconomic status, family history and systemic diseases, or history of atypical infection, all of which could be considered risk factors for uveitis. Third, the database data began in early 1996, but the data is incomplete. Therefore, we only analyzed the longitudinal data between the beginning of 2000 and the end of 2010. In order to avoid subjects being mistakenly diagnosed or mistakenly coded by accident as uveitis cases. We therefore defined patients with at least two times from principal/secondary diagnoses in outpatient visits and/or one times hospitalizations to ensure the validity of diagnosis. However, this is not equivalent to medical records. Therefore, the ICD-9 codes used in this study were restricted to non-infectious uveitis, mainly autoimmune uveitis. Previous studies used the positive predictive value (PPV) of the ICD-9 code to identify uveitis, and notes that PPV is higher than 80% of uveitis codes (38). To the best of our knowledge, the epidemiology of infectious uveitis is a complex relationship between microorganisms and humans. The etiology of infectious uveitis varies greatly (39, 40). In addition, this study cannot determine the validity of the diagnosis. However, we found that the stability of the diagnosis of uveitis was determined by our selection criteria, and the results were acceptable. Our goal was to determine the overall risk of various comorbidities in the uveitis population and, thus, we did not consider the subjects’ drug mixed factors. Therefore, a possible bias might exist.

## Conclusion

In conclusion, this retrospective cohort study demonstrated the association between air pollution and the risk of uveitis. In particular, those living in urbanization level 1 areas were exposed to a Q4 level of pollutants, which further increased the risk of uveitis. Further studies are required to investigate whether less exposure to pollutants can decrease the incidence of uveitis, and to examine the underlying mechanism.

## Data Availability Statement

The original contributions presented in the study are included in the article/**Supplementary Material**. Further inquiries can be directed to the corresponding author.

## Ethics Statement

The studies involving human participants were reviewed and approved by MOHW109-TDU-B-212-114004. Written informed consent for participation was not required for this study in accordance with the national legislation and the institutional requirements.

## Author Contributions

YB and CW: Study design and manuscript preparation. CL: statistical analysis. JW and JL: critical comment and revision. All authors contributed to the article and approved the submitted version.

## Conflict of Interest

The authors declare that the research was conducted in the absence of any commercial or financial relationships that could be construed as a potential conflict of interest.
