# Supplementary material for: Association Between Air Pollution and the Risk of Uveitis: A Nationwide, Population-Based Cohort Study
Source: Front Immunol. 2021 Mar 18;12:613893. doi: 10.3389/fimmu.2021.613893 (PMC8013994; doi:10.3389/fimmu.2021.613893)
Supplement: Supplementary file 1 [file DataSheet_1.docx]

**Supplement Table 1**. The risk of Uveitis in patients exposed to various air pollutants stratified by quartile of daily average concentration by IDW methods (sensitivity analysis) in Cox proportional hazard regression.

|  | | Event | IR | cHR | (95%CI) | aHR† | (95%CI) |
| --- | --- | --- | --- | --- | --- | --- | --- |
|  | **Carbon monoxide (CO)** | | | | | | |
| Quartile 1, <0.45 ppm | | 398 | 0.76 | Reference group | | Reference group | |
| Quartile 2, 0.45-0.54 ppm | | 256 | 0.65 | 0.85 | (0.73-1.00) | 0.98 | (0.77-1.06) |
| Quartile 3, 0.54-0.66 ppm | | 457 | 0.73 | 0.95 | (0.83-1.09) | 1.04 | (0.90-1.19) |
| Quartile 4, >0.66 ppm | | 1,097 | 2.28 | 2.98 | (2.66-3.34)*** | 3.09 | (2.75-3.46)*** |
|  | **Nitric oxide (NO)** | | | | | | |
| Quartile 1, <5.01 ppb | | 500 | 0.95 | Reference group | | Reference group | |
| Quartile 2, 5.01-7.29 ppb | | 349 | 0.69 | 0.72 | (0.62-0.83)*** | 0.76 | (0.66-0.87)*** |
| Quartile 3, 7.29-13.6 ppb | | 558 | 1.19 | 1.25 | (1.10-1.41)*** | 1.34 | (1.18-1.51)*** |
| Quartile 4, >13.6 ppb | | 801 | 1.53 | 1.61 | (1.44-1.80)*** | 1.73 | (1.54-1.93)*** |
|  | **Nitrogen oxides (NOx)** | | | | | | |
| Quartile 1, <21.9 ppm | | 476 | 0.91 | Reference group | | Reference group | |
| Quartile 2, 21.9-28.8 ppm | | 394 | 0.78 | 0.86 | (0.75-0.98)* | 0.92 | (0.80-1.05) |
| Quartile 3, 28.8-36.4 ppm | | 436 | 1.08 | 1.18 | (1.04-1.35)* | 1.25 | (1.09-1.42)*** |
| Quartile 4, >36.4 ppm | | 902 | 1.51 | 1.65 | (1.47-1.84)*** | 1.81 | (1.61-2.02)*** |
|  | **Total hydrocarbon (THC)** | | | | | | |
| Quartile 1, <10.3 ppm | | 108 | 0.26 | Reference group | | Reference group | |
| Quartile 2, 10.3-21.9 ppm | | 174 | 0.39 | 1.49 | (1.17-1.90)** | 1.48 | (1.16-1.88)** |
| Quartile 3, 21.9-27.3 ppm | | 744 | 2.43 | 9.24 | (7.55-11.3)*** | 8.71 | (7.12-10.6)*** |
| Quartile 4, >27.3 ppm | | 1,182 | 1.37 | 5.22 | (4.28-6.36)*** | 4.94 | (4.05-6.01)*** |
|  | **Methane (CH4)** | | | | | | |
| Quartile 1, <8.53 ppm | | 115 | 0.23 | Reference group | | Reference group | |
| Quartile 2, 8.53-17.1 ppm | | 213 | 0.58 | 2.49 | (1.98-3.12)*** | 2.48 | (1.97-3.11)*** |
| Quartile 3, 17.1-23.3 ppm | | 687 | 2.13 | 9.09 | (7.46-11.0)*** | 8.64 | (7.09-10.5)*** |
| Quartile 4, >23.3 ppm | | 1,193 | 1.41 | 6.05 | (5.00-7.33)*** | 5.74 | (4.74-6.95)*** |

IR, incidence rate (per 1,000 person-years)

cHR, crude hazard ratio; aHR, adjusted hazard ratio; CI, confidence interval

The daily average air pollutant concentrations were categorized into 4 groups based on quartiles for each air pollutant.

^†^Adjusted for age, sex, urbanization level, and comorbidities, including diabetes mellitus, hypertension, hyperlipidemia, asthma, COPD, psoriatic diseases, rheumatoid arthritis, SLE, and Behcet’s syndrome.

*p<0.05, **p<0.01, ***p<0.001

**Supplement Table 2.** Differences in Uveitis incidences and associated HRs in participants exposed to daily average concentrations of CO, NO, NOx, THC and CH4 stratify by follow-up period.

| Pollutant levels, unit | cHR(95%CI) | aHR(95%CI) | cHR(95%CI) | aHR(95%CI) |
| --- | --- | --- | --- | --- |
|  | Follow-up period ≦5 | | Follow-up period >5 | |
| CO |  |  |  |  |
| <0.61, per ppm | 1.00 | 1.00 | 1.00 | 1.00 |
| 0.61-0.76 per ppm | 1.00(0.82, 1.21) | 1.08(0.89, 1.31) | 0.95(0.81, 1.10) | 1.01(0.87, 1.17) |
| >0.76 per ppm | 2.91(2.48, 3.41)*** | 3.23(2.74, 3.81)*** | 1.65(1.44, 1.88)*** | 1.72(1.49, 1.98)*** |
| NO |  |  |  |  |
| <6.54 per ppb | 1.00 | 1.00 | 1.00 | 1.00 |
| 6.54-11.1 per ppb | 1.18(0.99, 1.40) | 1.31(1.09, 1.56)** | 1.33(1.15, 1.54)*** | 1.41(1.21, 1.63)*** |
| >11.1 per ppb | 1.89(1.62, 2.20)*** | 2.14(1.82, 2.53)*** | 1.39(1.21, 1.60)*** | 1.44(1.24, 1.67)*** |
| NOx |  |  |  |  |
| <27.1 per ppm | 1.00 | 1.00 | 1.00 | 1.00 |
| 27.1-36.4 per ppm | 1.30(1.09, 1.55)** | 1.43(1.20, 1.70)*** | 1.21(1.05, 1.40)*** | 1.30(1.12, 1.51)*** |
| >36.4 per ppm | 2.27(1.94, 2.66)*** | 2.51(2.13, 2.96)*** | 1.49(1.29, 1.70)*** | 1.53(1.33, 1.77)*** |
| THC |  |  |  |  |
| <2.33 per ppm | 1.00 | 1.00 | 1.00 | 1.00 |
| 2.33-2.52 per ppm | 9.24(5.78, 14.8)*** | 1.77(1.47, 2.12)*** | 1.77(1.47, 2.12)*** | 1.73(1.44, 2.07)*** |
| >2.52 per ppm | 18.3(11.6, 29.1)*** | 2.02(1.68, 2.42)*** | 2.02(1.68, 2.42)*** | 2.05(1.70, 2.47)*** |
| CH4 |  |  |  |  |
| <2.02 per ppm | 1.00 | 1.00 | 1.00 | 1.00 |
| 2.02-2.09 per ppm | 6.27(3.82, 10.3)*** | 6.37(3.89, 10.5)*** | 1.37(1.13, 1.65)** | 1.39(1.15, 1.69)*** |
| >2.09 per ppm | 22.7(14.1, 36.3)*** | 21.3(13.3, 34.2)*** | 2.43(2.04, 2.90)*** | 2.41(2.02, 2.87)*** |

IR, incidence rate (per 1000 person-years)

cHR, crude hazard ratio; aHR, adjusted hazard ratio; CI, confidence interval

The daily average air pollutant concentrations were categorized into 3 groups based on tertile for each air pollutant.

^†^Adjusted for age, sex, urbanization level, and comorbidities, including diabetes mellitus, hypertension, hyperlipidemia, asthma, COPD, psoriatic diseases, rheumatoid arthritis, SLE, and Behcet’s syndrome.

*p<0.05, **p<0.01, ***p<0.001
